# Supplementary material for: Tick-Tock Consider the Clock: The Influence of Circadian and External Cycles on Time of Day Variation in the Human Metabolome—A Review
Source: Metabolites. 2021 May 19;11(5):328. doi: 10.3390/metabo11050328 (PMC8161100; doi:10.3390/metabo11050328)
Supplement: Supplementary file 1 [file metabolites-11-00328-s001.zip › Supplementary materials/Supplementary figures.pdf]

% of Significantly Gradient Metabolites per Sample Type, Morning *vs* Evening Studies

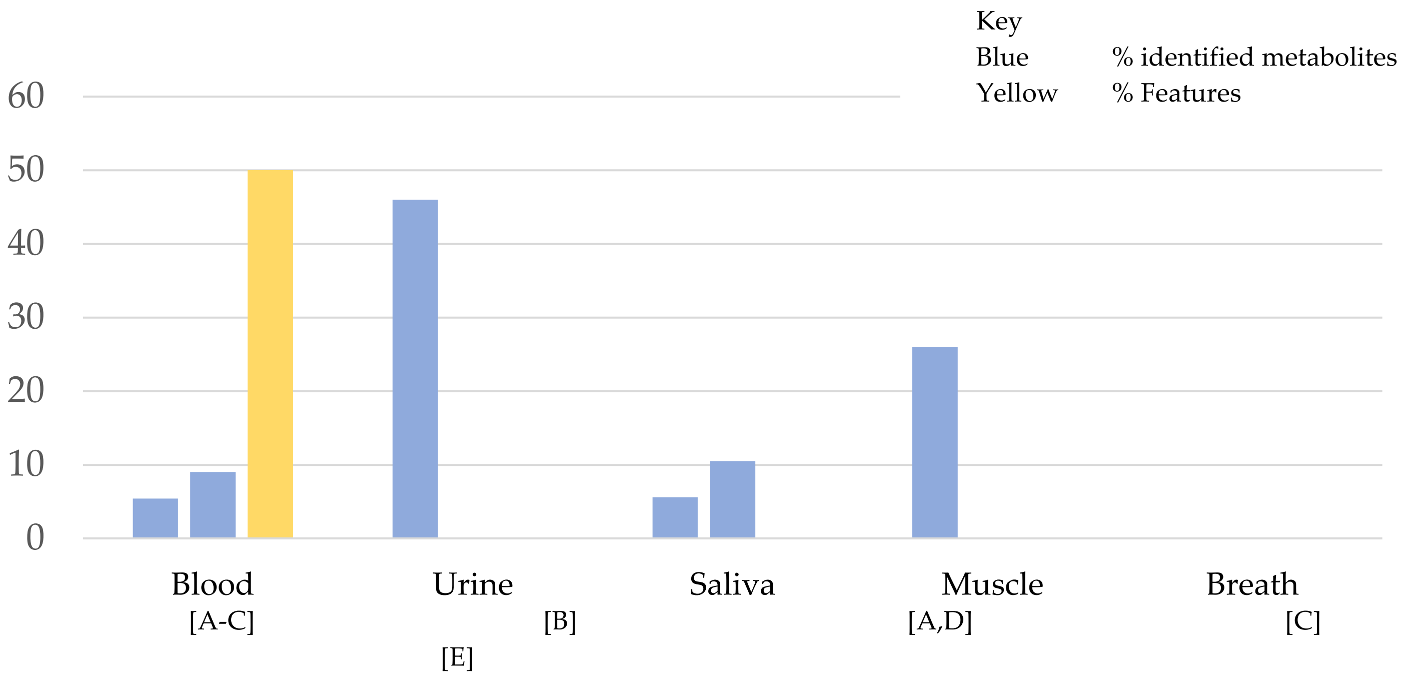

Figure S1: Percentage of observed metabolites/features undergoing a significant change between morning sample(s) and evening sample(s) as a function of rhythmic metabolites(features)/total metabolites(features).

Bars are indexed left to right

A. [68]
B. [67]
C. [66]
D. [76]
E. [77]

Note: Total number of features observed in Sinues [76] study was not disclosed thus a % not calculated. The study and 'Breath' section is still presented to illustrate that such a study has taken place.

Data from [60,70,71] excluded as the total observed metabolites for these studies was not disclosed.

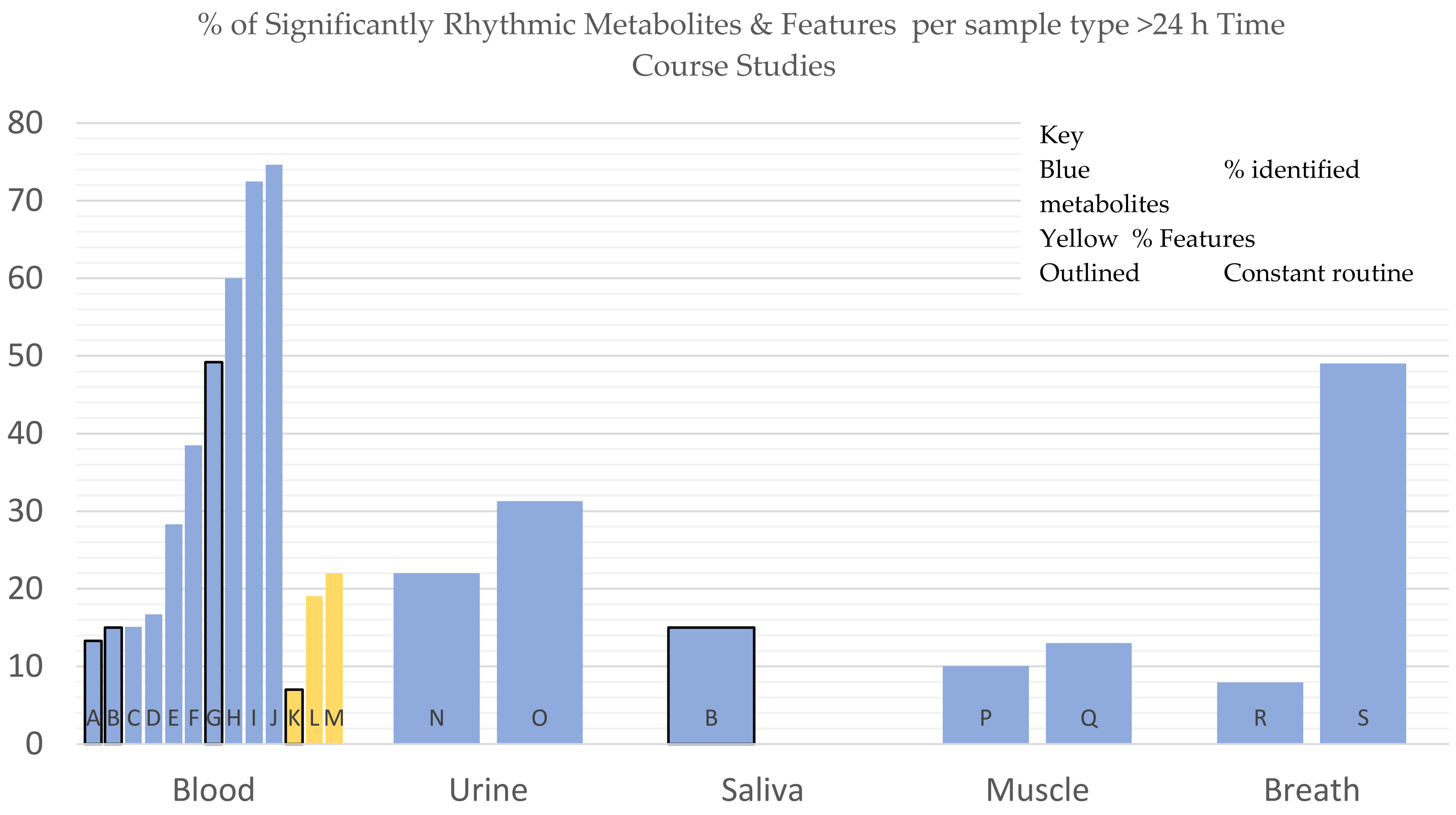

Figure S2: Percentage of observed significantly rhythmic metabolites and features as a function of rhythmic/total

Bars are indexed left to right

A. [46]
B. [37]
C. [61]
D. [63]
E. [54]
F. [56]
G. [58]
H. [59]
I. [52]
J. [55]
K. [45]
L. [57]
M. [54]
N. [72]
O. [73]
P. [81]
Q. [80]
R. [79]
S. [78]

The quoted % of rhythmic metabolites reflects the number of rhythmic metabolites of the total observed across any and all conditions tested, specific individual test groups therefore observed less than the % shown here for the following studies: Davies, Isherwood, Skene, Gu, Kervezee, Honma, Lusczek, for a more detailed breakdown please see Table 2

Jerjes [69] was excluded due to primarily focussing on cortisol and its derivatives, markers of rhythmicity, and would skew the % of observed rhythmicity in urine.

Data from Gehrman [62] excluded as the total observed metabolites for these studies was not disclosed.
